# The association between problematic social networking site use, dark triad traits, and emotion dysregulation

**DOI:** 10.1186/s40359-021-00668-6

**Published:** 2021-10-18

**Authors:** Zaheer Hussain, Elisa Wegmann, Mark D. Griffiths

**Affiliations:** 1 School of Social Sciences, Nottingham Trent University, Nottingham, UK; 2 General Psychology: Cognition and Center for Behavioral Addiction Research (CeBAR), University of Duisburg-Essen, Duisburg, Germany; 3 International Gaming Research Unit, School of Social Sciences, Nottingham Trent University, Nottingham, UK

**Keywords:** Problematic social networking site use, Machiavellianism, Narcissism, Psychopathy, Emotion dysregulation

## Abstract

**Background:**

Social networking sites (SNSs) allow people to socially connect with each other, collaborate, and share information. However, problematic SNS use (PSNSU) may be associated with negative personality traits. The present study investigated the associations between PSNSU, dark triad personality traits, and emotion dysregulation.

**Method:**

In the present study, 555 SNS users (*M*_*age*_ = 33.32 years, *SD* = 10.88) completed an online survey comprising measures of PSNSU, dark triad personality traits, and emotion dysregulation.

**Results:**

Bivariate correlations showed that PSNSU was significantly associated with dark triad traits as well as emotion dysregulation. Structural equation modelling (where the effect of the dark triad traits on PSNSU was mediated by emotion dysregulation) showed that 33.5% of the variance of PSNSU was explained by Machiavellianism, psychopathy, and narcissism.

**Conclusion:**

The findings provide suggestive evidence of why PSNSU may occur as a function of the presence of dark triad traits and emotion dysregulation. The study also highlighted the important role that emotion regulation plays in the association between dark triad traits and PSNSU.

## Background

Billions of people now have multiple international social connections courtesy of social networking sites (SNSs). Research suggests that 24% of teenagers use SNSs almost constantly and 71% use more than one SNS [1]. There are many benefits of using SNSs. They (1) elevate the ease in which individuals may form and create online communities [2], (2) improve collaboration and sharing of information [3], (3) can lead to the creation of new job roles [4], (4) allow users to be constantly connected to friends, (5) allow for ease of communication, information transfer, and (6) help break down social boundaries [5].

However, the negative consequences of SNS use cannot be ignored. Problematic social networking site use (PSNSU, and in extreme cases ‘SNS addiction’) are examples of such negative consequences. Andreassen and Pallesen [6] have defined PSNSU as being preoccupied with SNSs, having a strong motivation to use SNSs, and spending excessive amounts of time on SNSs leading to impairments in social, personal and/or professional life, as well as psychological health and wellbeing. Unlike other addictive disorders, such as gaming disorder [7], PSNSU has not yet been recognized as a clinical disorder and researchers recommend discussing the problematic behaviour against the background of guidelines such as (1) the presentation of clinical relevance, (2) a theoretical embedding, and (3) a better understanding of underlying mechanisms [8] when considering PSNSU as potential “other specified disorders due to addictive behaviours”.

Regarding the clinical relevance, some research studies highlight prevalence rates of individuals suffering from problematic use (e.g., [9, 10]) with one study [11] reporting that 29.5% of the sample experienced negative consequences due to PSNSU. Bányai et al. [12] showed in one nationally representative study that among nearly 6000 Hungarian teenagers, 4.5% reported to be at risk of developing a PSNSU [12]. However, the lack of classification as well as non-standardized diagnostic or assessment procedures make the comparability across different studies difficult.

Regarding the theoretical embedding of PSNSU, several theoretical approaches from addiction research have been used to identify and to explain key factors and underlying mechanisms of the development and maintenance of a potential addictive behaviour, for example the dual-process approach [13, 14] as well as the idea of cue-reactivity and craving as key factors of an addictive behaviour [15, 16, 17]. However, there are some theoretical approaches focusing on problematic Internet use in general as well as on PSNSU in specific. The I-PACE (Interaction of Person-Affect-Cognition-Execution) model by Brand et al. [18] and its updated version [19] describes the process involved in the development and maintenance of a potential addictive (online) behaviour. The model outlines that specific predisposing variables such as personality traits, using motives, social cognitions, and psychopathological symptoms affect the perception of internal and external triggers. It is also assumed that the perception of those triggers results in specific affective and cognitive responses, which are also affected by subjective coping style, emotion regulation strategies, and inhibitory control leading to the decision to use a specific online application, for example SNSs. Based on reward-related learning and reinforcement processes, it is assumed to result in a continuation of the behaviour, whereas individuals experience a diminished control and negative consequences in daily life [19].

In addition to many components of the model, a key assumption is that the effect of individual personal characteristics on a specific behaviour is reinforced and/or mediated by affective and cognitive components, but also by difficulties in regulating emotions and in dealing with conflicts illustrated as dysfunctional coping style. Empirical findings already emphasized that the effect of personality traits, social cognitions, and psychopathological symptoms on tendencies towards an Internet-use disorder in general as well towards PSNSU was mediated by dysfunctional coping [20, 21]. Other theoretical models, such as the social skills model [22, 23] and the cognitive-behavioural model [24], emphasise the role of maladaptive cognitions, the effects of psychopathology, and psychosocial problems on the formation of problematic online behaviours. In line with this, [25] also argues that the Internet is used for compensation of deficits and as a specific coping strategy resulting in overuse. He stressed that predisposing variables, usage motives, and environmental factors should be considered when investigating the relation of dysfunctional coping and Internet use.

Moreover, the theoretical assumptions by Wegmann and Brand [26] emphasize that PSNSU is mainly associated with psychosocial characteristics that drive the behaviour in order to experience gratification of specific needs or compensation of deficits. The authors propose two pathways: the fear-driven/compensation seeking hypothesis and the reward-driven hypothesis specified by specific needs, for example, the need to belong, and personality traits, narcissism, and reinforcement processes such as fear of missing out 26. Since the model is not exhaustive, it could also be assumed that further mediating factors such as emotion regulation strategies to experience rewards or to compensate for deficits also represent a reinforcing mechanism.

### The relevance of maladaptive personality traits on PSNSU

Studies investigating the associations between SNS use and personality as predisposing factors have demonstrated different personality traits to be associated with PSNSU [27–34]. However, in addition to these ‘functional’ traits, there are further studies addressing maladaptive or mainly ‘dysfunctional’ personality traits, which appear to be associated with problematic use of the internet (generally) and SNSs (more specifically). The anti-social traits commonly referred to as the dark triad (i.e., Machiavellianism, psychopathy, narcissism) and the dark tetrad (dark triad traits plus sadism) are now increasingly being researched [35–37]. Sindermann et al. [38] investigated the association between dark triad traits and different forms of problematic use of internet applications. They reported that symptoms of problematic internet use were related to Machiavellianism and psychopathy. For both males and females, psychopathy was positively associated with internet pornography use and problematic internet use in general. However, the correlation patterns differ with respect to preferred internet applications such as PSNSU, problematic online buying/shopping behaviour, and problematic pornography use. The authors concluded that the relationship between these traits and problematic online behaviour appear to be more complex and should be investigated in more detail. Kircaburun et al. [39] also reported that Machiavellianism and narcissism were positively associated with PSNSU, and males had higher scores on dark triad traits. Recently, research has reported associations between Machiavellianism, psychopathy and PSNSU [40, 41].

### The relevance of emotion dysregulation on PSNSU

Emotion regulation is a trait that refers to the process involved in monitoring and evaluating emotion in order to accomplish one’s goals [42]. Research offers support for the associations between difficulties in emotion regulation, mental health problems, and maladaptive behaviours [43, 44]. Emotion regulation difficulties have been shown to be related to substance use [45, 46], deliberate self-harm [47], risky sexual behaviour [48], and depression [49].

More recently, researchers have begun investigating the associations between difficulties in emotion regulation and addictive behaviours. Hormes et al. [50] examined PSNSU and its associations with difficulties with emotion regulation and substance use. The results showed that PSNSU appeared to arise due to poor emotion regulation skills and heightened susceptibility to both substance and non-substance-use disorder (as was also found in a study by Estévez et al. [45]). Elhai et al. [51] monitored daily smartphone use over the course of one week among 68 college students. Lower depression severity predicted increased smartphone use over the one-week period. Greater use of expressive suppression as an emotion regulation strategy predicted more baseline smartphone use, but less smartphone use during the one-week period.

More generally, several studies have investigated emotion regulation and problematic internet use with findings revealing that emotion regulation negatively correlated with PIU [45, 52, 53]. Taken together, research findings emphasize the relevance of emotion regulation strategies for investigating addictive use of internet applications as well as its interaction with predisposing variables. Further investigating the impact of emotion regulation could potentially help in the development of interventions to tackle PSNSU.

### Aims of the present study

Theoretical assumptions as well as empirical evidence emphasize that in addition to direct effects of personality traits on PSNSU, the relationship appears to be mediated by further cognitive and affective responses. Brand et al. [19], as well as Wegmann and Brand [26], illustrate the importance of predisposing factors such as personality traits resulting in a potential addictive behaviour such as PSNSU. However, the authors also highlight that besides those predisposing risk factors, further affective and cognitive responses as well as coping mechanisms such as emotion regulation abilities could mediate the relationship between vulnerable factors and PSNSU. It has been noted that dysfunctional coping strategies and emotion dysregulation could result in repeated usage of specific online applications for experiencing the gratification of specific needs or to compensate for emotional or social deficits [18, 19, 21].

**Fig. 1:**
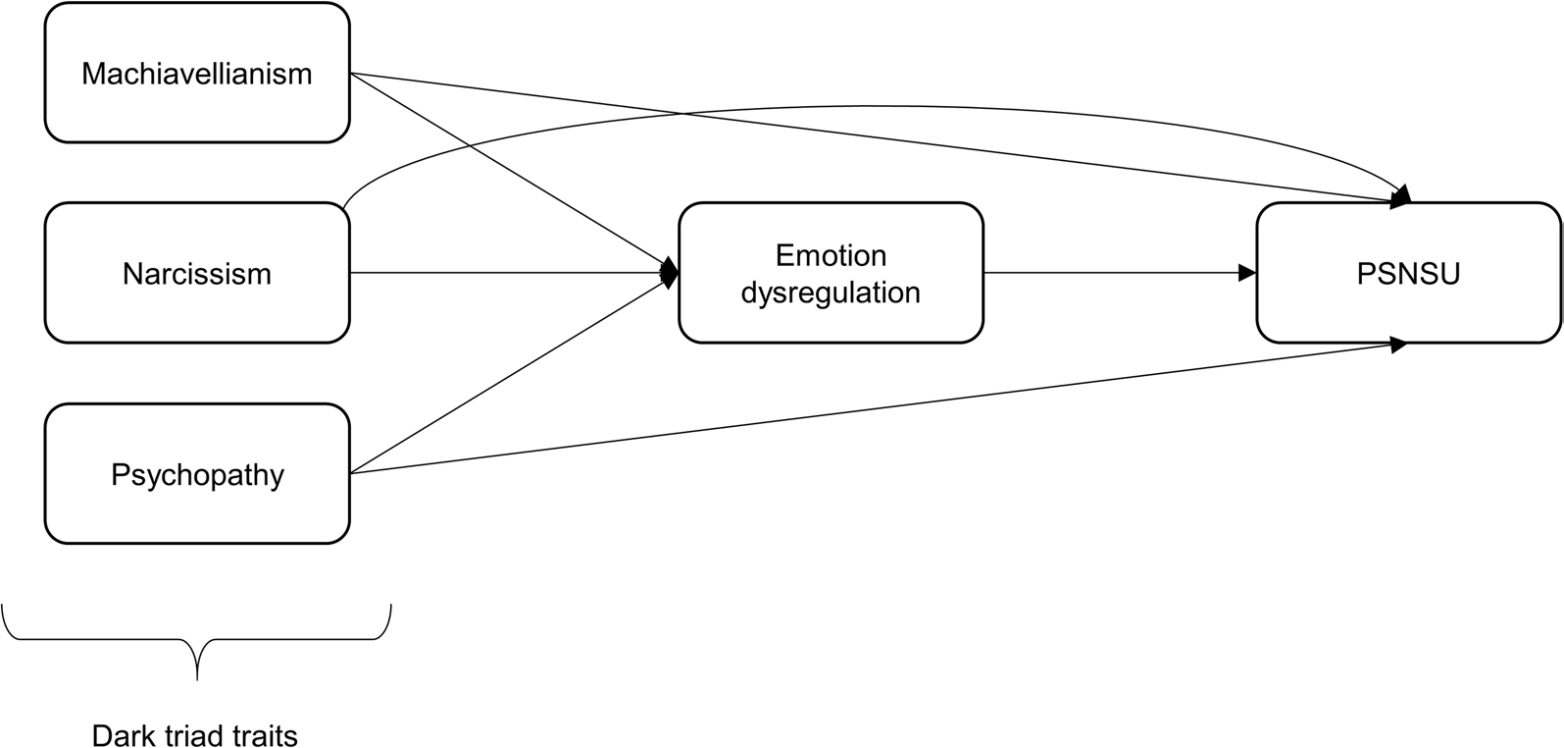
The operationalised model for analysing the main assumptions with PSNSU as dependent variable

Moreover, research outlines that this relationship can be observed as direct and indirect effects of dark triad traits on PSNSU [39, 54]. Consequently, in addition to the direct effect of personality traits, and based on these aforementioned theoretical as well as empirical considerations, it could be assumed that emotion regulation strategies or emotion dysregulation play an important role in the development and maintenance of internet use disorders (such as PSNSU). Understanding the process in the development and maintenance of PSNSU is an important first step. Knowledge concerning reinforcement mechanisms would be useful for defining preventive factors as well as being of use in treatment programs for individuals suffering from negative consequences. Therefore, the present study examined the associations between PSNSU, dark triad personality traits, and emotion dysregulation. Emotion dysregulation was tested as a mediator due to previous research showing that it may be a key variable in managing problematic behaviour. The hypothesized mediation model is shown in Fig. 1.

## Method

### Participants

The present study comprised 555 participants (264 females, 291 males) aged between 18 and 80 years (*M* = 33.32 years, *SD* = 10.88). Initially, there were 566 survey responses, but following a reliability check question (where participants were asked to answer the question “I breathe oxygen each day”, and only the individuals who gave a correct response to this question were included), 555 valid and complete responses remained for data analysis. Of these, 35 participants were students, 389 were employed, 76 were self-employed, 42 were unemployed, and 13 were retired.

Since SNS usage was the focus of the present study, participants were also asked which SNS platform they preferred the most. The most preferred sites were Facebook (59.64%), Instagram (10.45%), YouTube (7.38%), WhatsApp Messenger (7.38%), Twitter (6.48%) and others (less than 5%). Regarding their preferred social networking activities, responses included checking the news feed (27.93%), viewing photos (19.46%), chatting to friends (16.22%), and updating their status (11.35%). Participants indicated spending one to 980 min each day on SNSs (*M* = 118.88 min, *SD* = 102.36).

### Design and materials

A cross-sectional survey design was utilized in the present study. Several measures were used to investigate the study variables. These measures are described below.

#### Bergen Social Media Addiction Scale

The Bergen Social Media Addiction Scale (BSMAS), a modified version of the Bergen Facebook Addiction Scale (BFAS; [29]) was used to assess PSNSU. Scale questions were modified by Andreassen et al. [55] by substituting the word ‘social media’ for the word ‘*Facebook*’ and has been validated in a number of studies (e.g., [12, 56–58]). Six items assess the six core criteria of addiction outlined by Griffiths [59], i.e., salience, conflict, mood modification, withdrawal, tolerance, and relapse. Responses are provided on a five-point Likert scale ranging from 1 (*very rarely*) to 5 (*very often*). Total scores are obtained by summing participant ratings of each item, with higher scores indicating higher PSNSU severity. The mean BSMAS score ranges from 1 to 5 and the internal reliability of the scale in the present study was good (α = 0.789). The BSMAS was used as a continuous variable similar to previous research studies [60, 61, 62].

#### Short Dark Triad Scale

The Short Dark Triad Scale (SD3; [63]) was used to assess the dark triad personality traits of Machiavellianism, narcissism, and psychopathy. The SD3 is a 27-item measure with five-point Likert responses ranging from 1 (*disagree strongly*) to 5 (*agree strongly*). Example items include ‘it’s not wise to tell your secrets’, ‘people see me as a natural leader’, and ‘it’s true that I can be mean to others’. The mean of the items within each subscale was calculated with higher mean scores indicating higher levels of dark triad traits. Each of the three subscales comprises nine items, and the internal reliabilities of all subscales were good to very good (Machiavellianism α = 0.834; narcissism α = 0.745; psychopathy α = 0.848).

#### Difficulties in Emotion Regulation Scale

The short version of the Difficulties in Emotion Regulation Scale (DERS-16; [64]) was used to assess difficulties in emotion regulation. The DERS-16 is a self-report measure that assesses individuals’ typical level of difficulties in emotion regulation. The scale comprises 16 items, example items include ‘When I’m upset, I feel ashamed with myself for feeling that way’, ‘When I’m upset, I have difficulty getting work done’, ‘When I’m upset, I feel out of control’, and ‘I have difficulty making sense out of my feelings’. Respondents rate the extent to which each item applies to them on a 5-point Likert-type scale from 1 (*almost never*) to 5 (*almost always*). Besides the overall mean score of the DERS-16, further mean values of the following subscales are calculated: lack of emotional clarity (two items), difficulties engaging in goal-directed behaviour (three items), impulse control difficulties (three items), non-acceptance of emotional responses (three items), and limited access to effective emotion regulation strategies (four items). Higher values reflect greater levels of emotion dysregulation. The internal reliability of the overall mean score in the present study was excellent (α = 0.927). The five subscales of the questionnaire are used to represent the latent dimension of “emotional dysregulation” in the structural equation model.

### Procedure

A recruitment message was posted on the online crowdsourcing website *Amazon Turk* inviting SNS users aged 18 years or older to participate in the study. The recruitment phase lasted seven days. The online recruitment post included information about the purpose of the study and a hyperlink to the online survey. The hyperlink directed participants to the survey where they were presented with a participant information page followed by clear instructions on how to complete the survey. All participants provided written consent by ticking a box in the online survey that asked them to acknowledge consent to participate. All participants were assured that their data would remain anonymous and confidential. A debriefing statement at the end of the survey reiterated the purpose of the study and informed participants of their right to withdraw from the study. Participants were paid for survey completion through the *Amazon Turk* online interface.

### Ethics

The study was carried out in accordance with the Declaration of Helsinki and British Psychological Society Ethical Guidelines. The first author’s university ethics committee approved the study. All participants were informed about the study and all provided informed consent.

### Statistical analysis

Statistical analyses were carried out using SPSS 25.0 for Windows (IBM SPSS Statistics). Pearson's correlations were calculated to test bivariate relationships between variables. The structural equation model to test the aforementioned mediation effects were analysed using Mplus 6 [65]. Besides the mean scores of the variables mentioned we used the method of item parcelling. This method reduces the risk of measurement errors in structural equation models [66, 67]. Therefore, we split the items of the BSMAS and the subscales of the SD3 in two half and calculated two further mean scores from half of the variables each. We then had additional sub-factors, which had been used to define the latent dimensions of the predictors (narcissism, Machiavellianism, and psychopathy), as well as the depended variable PSNSU by using two sub-factors each. For the latent dimension emotion dysregulation, no item parcelling was needed since the questionnaires used already represented the construct by all five sub-factors (as described in the Methods section).

The evaluation of the model fit was done with standard criteria: standardized root mean square residual (SRMR; values < 0.08 indicated a good fit with the data), comparative fit indices (CFI/TLI; values > 0.90 indicated an acceptable fit with the data), and root mean square error of approximation (RMSEA; values < 0.08 indicated a good fit with the data) [68, 69]. Additionally, the χ^2^ test was used to check the data derivation of the defined model. However, before analysing the structural equation model all relevant variables were checked to see if they correlated with each other [70].

## Results

### Descriptive values and correlation analyses

The descriptive values such as the mean scores and the standard deviations for all scales and the additional sub-factors defined by the method of item parcelling as well as the bivariate correlations between all aforementioned variables are shown in Table 1. The results showed that PSNSU overall score was significantly associated with dark triad traits and emotion dysregulation. Emotion dysregulation was also significantly correlated with dark triad traits. In addition, the subscales of emotion dysregulation as well as the sub-factors calculated by item parcelling also significantly correlated.

**Table 1:** Mean scores, standard deviations and bivariate correlations between the aforementioned variables

	*M *(*SD*)	Age^+^	2	2a	2b	3	3a	3b
1. PSNSU (Overall score)	2.50 (0.80)	− .237**	.343**	.351**	.287**	.294**	.333**	.163**
1a PSNSU sub-factor 1	2.57 (0.87)	− .195**	.298**	.310**	.244**	.246**	.288**	.123**
1b PSNSU sub-factor 2	2.42 (0.87)	− .240**	.333**	.336**	.285**	.296**	.326**	.177**
2. Machiavellianism (Overall score)	3.39 (0.72)	− .275**		.949**	.927**	.545**	.583**	.352**
2a Machiavellianism sub-factor 1	3.39 (0.75)	− .261**			.760**	.538**	.585**	.334**
2b Machiavellianism sub-factor 2	3.38 (0.78)	− .255**				.479**	.501**	.325**
3. Narcissism (Overall score)	2.98 (0.62)	− .245**					.933**	.847**
3a Narcissism sub-factor 1	3.11 (0.74)	− .237**						.599**
3b Narcissism sub-factor 2	2.82 (0.63)	− .196**						
4. Psychopathy (Overall score)	2.65 (0.83)	− .350**						
4a Psychopathy sub-factor 1	2.68 (0.89)	− .351**						
4b Psychopathy sub-factor 2	2.61 (0.87)	− .306**						
5. Emotion dysregulation (Overall score)	2.22 (0.78)	− .288**						
5a Lack of emotional clarity	2.01 (0.92)	− .240**						
5b Difficulties engaging in goal-directed behaviour	2.45 (0.95)	− .215**						
5c Impulse control difficulties	2.04 (0.94)	− .251**						
5d Non-acceptance of emotional responses	2.29 (0.96)	− .221**						
5e Limited access to effective emotion regulation strategies	2.23 (0.88)	− .271**						

	4	4a	4b	5	5a	5b	5c	5d	5e
1. PSNSU (Overall score)	.347**	.337**	.317**	.423**	.399**	.339**	.427**	.281**	.356**
1a PSNSU sub-factor 1	.241**	.235**	.219**	.382**	.325**	.329**	.374**	.261**	.325**
1b PSNSU sub-factor 2	.397**	.384**	.364**	.396**	.409**	.293**	.412**	.256**	.329**
2. Machiavellianism (Overall score)	.655**	.637**	.597**	.342**	.322**	.209**	.393**	.263**	.277**
2a Machiavellianism sub-factor 1	.599**	.578**	.552**	.317**	.298**	.195**	.361**	.256**	.250**
2b Machiavellianism sub-factor 2	.633**	.622**	.569**	.326**	.307**	.197**	.377**	.237**	.272**
3. Narcissism (Overall score)	.521**	.491**	.495**	.122**	.169**	− .008	.278**	.083	.049
3a Narcissism sub-factor 1	.554**	.527**	.522**	.149**	.201**	.001	.296**	.110**	.075
3b Narcissism sub-factor 2	.340**	.315**	.330**	.052	.079	− .018	.182**	.022	− .003
4. Psychopathy (Overall score)		.958**	.930**	.428**	.399**	.223**	.548**	.288**	.363**
4a Psychopathy sub-factor 1			.787**	.429**	.393**	.233**	.549**	.276**	.367**
4b Psychopathy sub-factor 2				.375**	.357**	− 183**	.479**	.268**	.313**
5. Emotion dysregulation (Overall score)					.719**	.832**	.817**	.831**	.929**
5a Lack of emotional clarity						.530**	.576**	.482**	.594**
5b Difficulties engaging in goal-directed behaviour							.567**	.609**	.730**
5c Impulse control difficulties								.589**	.683**
5d Non-acceptance of emotional responses									.727**
5e Limited access to effective emotion regulation strategies									

**p ≤ .010, ^+^control variable, *M* mean values, *SD* standard deviation

### The structural equation model

**Fig. 2:**
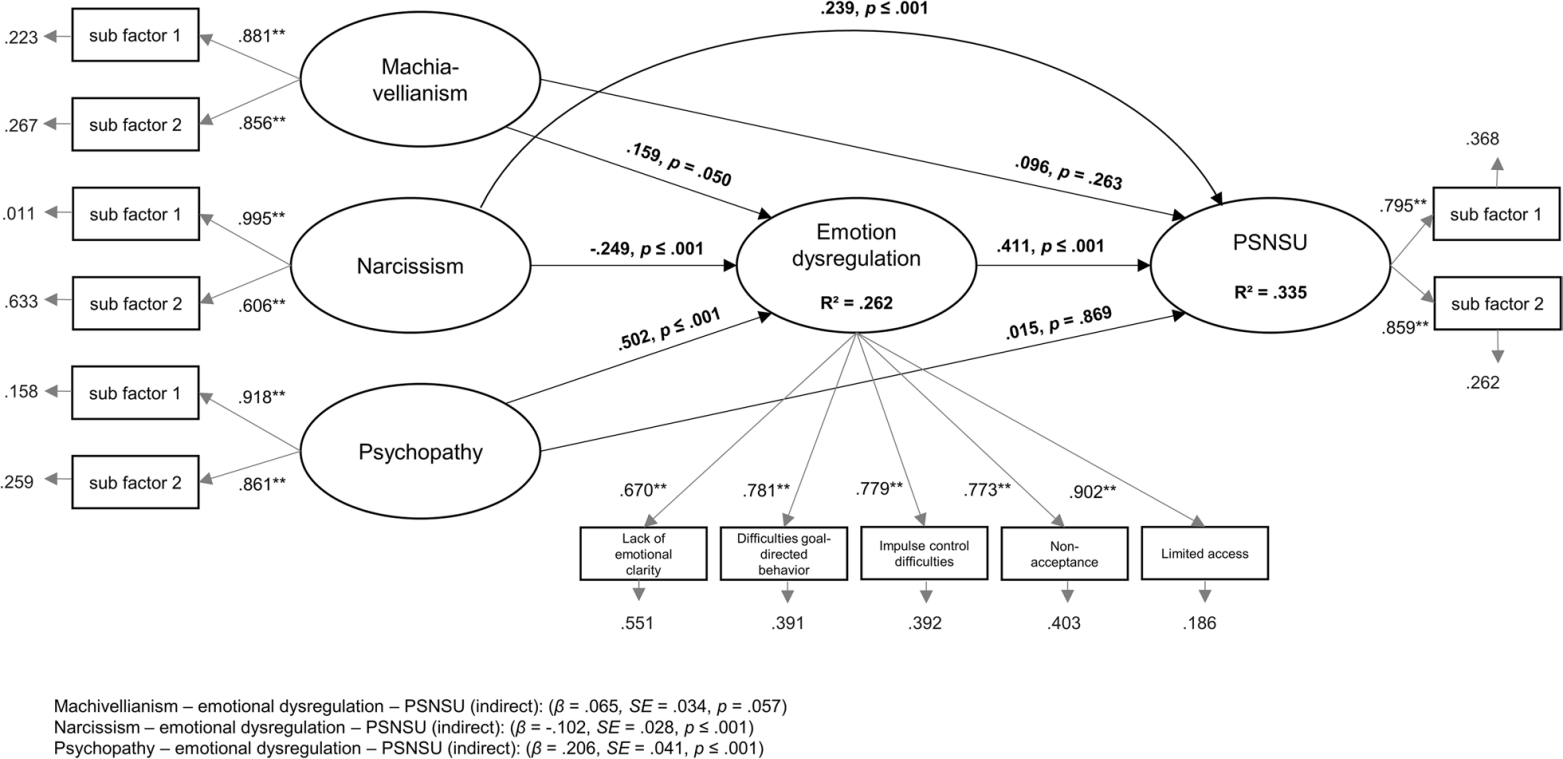
Results of the structural equation model including factor loadings on the described variables and the accompanying *β*-weights, *p* values, residuals, and indirect effects

In the structural equation model, it was assumed that the effect of all dark triad traits (Machiavellianism, psychopathy, and narcissism) on PSNSU was mediated by emotion dysregulation. Based on the idea of item parcelling, we used the sub-factors of the BSMAS for the latent dimension *PSNSU*, the sub-factors of the dark triad subscales for the latent dimension *Machiavellianism*, *psychopathy*, and *narcissism*, and all subscales assessing emotion dysregulation for the latent dimension *emotion dysregulation*. The structural equation model with PSNSU as dependent variables showed an excellent fit with the data. The RMSEA was 0.086, *p* ≤ 0.01, CFI was 0.947, TLI was 0.924, and the SRMR was 0.056. The χ^2^ test was significant, (*p* ≤ 0.01). Overall, 33.5% of the latent dimension *PSNSU* variance was explained by the proposed model (*R*^*2*^ = 0.335, SE = 0.041, *p* ≤ 0.001). The structural equation model with the factor loading and the *β*-weights are shown in Fig. 2.

The results indicated that the latent dimensions were well represented by the manifest variables used. In addition, the results outlined that narcissism had a direct effect on PSNSU, while Machiavellianism and psychopathy had no direct effect on PSNSU. However, all dark triad traits had a direct effect on emotion dysregulation, which also had a direct effect on PSNSU. Additionally, the indirect effect of Machiavellianism on PSNSU mediated by emotion dysregulation was almost significant (*β* = 0.065, SE = 0.034, *p* = 0.057). The significant indirect effect of narcissism on PSNSU over emotion dysregulation (*β* = -0.102, SE = 0.028, *p* ≤ 0.001) indicated a partial mediation, whereas the significant effect of psychopathy on PSNSU over emotion dysregulation (*β* = 0.206, SE = 0.041, *p* ≤ 0.001) indicated a full mediation effect.

### Additional analyses

**Fig. 3:**
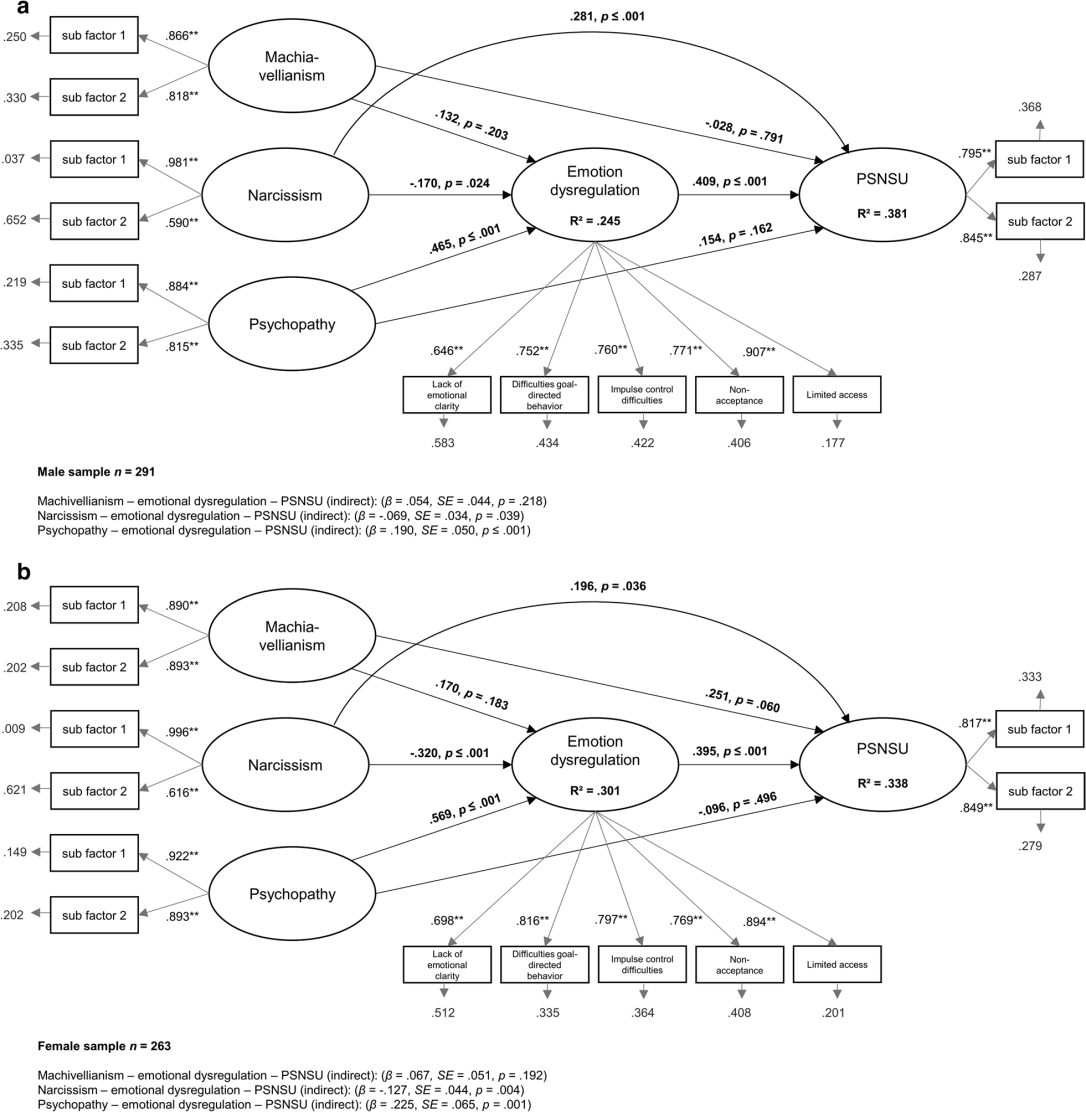
**a** Results of the structural equation model for the male sample including factor loadings on the described variables and the accompanying *β*-weights, *p* values, residuals, and indirect effects. **b** Results of the structural equation model for the female sample including factor loadings on the described variables and the accompanying *β*-weights, *p* values, residuals, and indirect effects

Empirical findings illustrate that male and female users, as well as younger users, differ in the development and maintenance of a PSNSU [12, 71, 72]. Therefore, demographic variables could have an effect on the structural equation model, which we would like to control for. At a first step, we calculated group comparison between male and female participants to check if there were significant gender differences in the aforementioned variables. The results showed that males had higher scores in dark triad personality traits compared to females (Table 2). For a better understanding of the underlying mechanisms of PSNSU, a further structural equation model was tested, which addressed gender differences in dark triad traits. Therefore, we ensured that the overall variables of the SEM significantly correlated with each other. For the male sample, all variables showed a significant correlation (*r* ≤ 0.439 and ≥ 0.309), except for emotion dysregulation and narcissism (*r* = 0.082). For the female sample, all variables significantly correlated with each other (*r* ≤ 0.459 and ≥ 0.152). The proposed model with additional differentiation by gender using mean structure analysis was analysed, which is often used to compare group means on the proposed constructs [73]. Again, the fit indices were good (RMSEA = 0.086, *p* ≤ 0.01, CFI = 0.937, TLI = 0.923, SRMR = 0.063). Both models, for males and females, are shown in Fig. 3a, b.

**Table 2:** Mean scores, standard deviations and group comparisons between male and female participants

	Male participants (*n* = 291)	Female participants (*n* = 263)	Group comparisons
PSNSU (Overall score)	14.74 (4.83)	15.27 (4.76)	*t*(553) = − 1.30, *p* = .194, *d* = 0.11
Machiavellianism (Overall score)	3.46 (0.68)	3.30 (0.75)	*t*(553) = 2.70, *p* = .007, *d* = − 0.22
Narcissism (Overall score)	3.08 (0.57)	2.87 (0.66)	*t*(520.44) = 3.89, *p* ≤ .001, *d* = − 0.34
Psychopathy (Overall score)	2.81 (0.71)	2.46 (0.92)	*t*(492.77) = 5.02, *p* ≤ .001, *d* = − 0.43
Emotion dysregulation (Overall score)	35.89 (11.99)	35.19 (13.07)	*t*(534.98) = 0.66, *p* = .510, *d* = − 0.06

*d* = Cohen’s *d* for effect size, *t* = *t*-value of the test statistics, *p* = *p*-value of the significance

For the male and female participants, the results showed a direct effect of psychopathy and emotion dysregulation on PSNSU. Machiavellianism and narcissism showed no direct effect. Narcissism and psychopathy had a direct effect on emotion dysregulation as well. In addition, the effect of psychopathy and narcissism on PSNSU was mediated by emotion dysregulation. Overall, 38.1% of the PSNSU variance was explained in the male sample (*R*^*2*^ = 0.381, *p* ≤ 0.001). In the female model sample, 33.8% of the PSNSU variance was explained (*R*^*2*^ = 0.338, *p* ≤ 0.001). In summary, the results showed similar direct and indirect effects for both males and females but when considering effect sizes and mediation patterns, there are some distinct gender differences. In a second step, the bivariate correlations were calculated between age and all other variables (see Table 1). The results showed significant effects with small to medium effect sizes. We additionally included age as a covariate in the structural equation model, however, the model fits showed no good fit with the data (RMSEA = 0.141, *p* ≤ 0.001, CFI = 0.827, TLI = 0.761, SRMR = 0.165). It could be assumed that the effect of age in PSNSU as well as in the development and maintenance of emotion regulation strategies might be based on different constructs. We recommend that the impact of age on PSNSU should be systematically addressed in future studies. Cognitive development along with the emotion regulation capacities may develop as the age increases. For instance, metacognition which represents a higher cognitive function has been found to be more salient than emotion recognition in adolescent PSNSU. On the other hand, emotion recognition difficulties have. been found to be prominent in adult PSNSU [74, 75]. Therefore, it is important to examine SNS tendencies in different age groups.

## Discussion

The present study investigated the associations between PSNSU, dark triad personality traits, and emotion dysregulation. Overall, 33.5% of the variance was explained in the assumed structural equation model. The results highlight the relationship between dark triad traits and PSNSU by indicating that maladaptive personality traits appear to be associated with PSNSU. Additionally, emotion dysregulation also appears to be associated with PSNSU. Furthermore, the effect of maladaptive personality traits was mediated by dysfunctional emotion regulation strategies, which appears to be a reinforcement mechanism and additional risk factor.

Individuals with maladaptive personality traits appear to be at higher risk for developing addictive behaviour, especially when they have impairments in regulating their own emotions. This appears to be the case for both male and female SNS users. Moreover, the results additionally show that the maladaptive personality traits differ in their effect on the development and maintenance of PSNSU. Since psychopathy and Machiavellianism did not predict symptom severity of PSNSU directly, narcissism could be identified as a direct risk factor. The direct effect of narcissism on PSNSU supports previous research by Malik and Khan [10] who reported that PSNSU was a significant predictor of narcissistic behaviour. This finding also lends support to previous research that has investigated narcissism and problematic online behaviour [76–78].

It could be concluded that individuals who have the desire to achieve admiration and self-enhancement such as a narcissist and those who show reduced empathy for the achievement of individual goals have higher difficulties in controlling their own SNS use. This could especially be the case in that most SNSs allow the presentation of an ideal self and the explicit control by users of their own impression management [79–82]. It might be assumed that this control and the desire of ideal self-presentation could become a potentially obsessive behaviour resulting in the urge to get positive feedback for their own profile or new status updates (e.g., [83]).

Recent research has shown that a specific dimension of grandiose narcissism (i.e., passive and dependent on others) is a risk factor for PSNSU, whilst the active and independent dimension could function as a protective factor [84], therefore not all aspects of the trait might contribute to addictive behaviour. Furthermore, a recent review study found that the association between vulnerable narcissism and PSNSU is under-researched [85]. This highlights the need for further research into the different dimensions of narcissism.

The mechanism for individuals with psychopathic and Machiavellian tendencies appear to be different. The bivariate correlations indicate a positive relationship between Machiavellianism, psychopathy, and PSNSU. However, the mediation effect illustrate that these relationships seem to be more complex. Individuals, who are impulsive, non-empathetic, selfish, manipulative, and ruthless (see [86]), seem to have difficulties in regulating their own emotion, which could lead to a higher vulnerability of developing PSNSU. These findings support previous research indicating that a dysfunctional coping style could be described as a reinforcement mechanism, which enhances the risk of problematic internet use when specific personality traits are investigated.

For example, Brand et al. [20] demonstrated that the effect of personality traits such as self-esteem, self-efficacy, and stress vulnerability on pathological internet use is mediated by a dysfunctional coping style. It emphasizes that difficulties in handling emotion as well as individual problems reinforce the effect of specific personality traits on addictive behaviour. Brand et al. [18, 19] assume that the relationship between predisposing variables and symptom severity of a potential addictive behaviour is reinforced by affective and cognitive mechanisms such as a dysfunctional coping style and dysfunctional emotion regulation. Similarly, the cognitive-behavioural model of IGD [87] argues that the interaction of cognitive components, decision making style, and motivational components explain problematic behaviour. Moreover, it could be assumed that dysfunctional emotion regulation strategies serve as reinforcing mechanisms for individuals to experience rewards or compensate (emotional) deficits by using SNSs [26].

This approach outlines that personality traits associated with problematic use of SNSs could be related to specific reinforcement mechanisms such as a more fear-driven or compensation seeking approach and a reward-driven pathway [88]. Besides the gratification of specific needs, fear of missing out, or compensation of (social) deficits as mediating factors, dysfunctional emotion regulation could be assumed as a further reinforcing factor. Based on the idea by Wegmann and Brand [26] the direct effect of narcissism on PSNSU illustrates a more reward-driven pathway. The disability of handling emotions maybe associated with a dysfunctional coping style as well could indicate a more fear-driven approach, where individuals with maladaptive personality traits try to compensate this dysfunctional emotion regulation strategy by using social networks resulting in a repeated behaviour and in problematic use at the end.

However, future research should investigate the effect of dysfunctional emotion regulation on PSNSU by keeping the fear-driven/compensation seeking hypothesis in mind. Nevertheless, the observation of very constant mediation effects emphasizes the importance of emotion regulation and the bidirectional association between dark triad traits and PSNSU. In terms of possible explanations, it shows that the more difficulties SNS users have in regulating their emotions the higher the chance of displaying PSNSU behaviour. The strategy used by individuals to manage their emotions could prove vital in preventing maladaptive SNS use. This could have implications for prevention and treatment programmes. It could be that specific content or interactions on SNSs causes individuals to experience negative emotions, encouraging individuals to be aware of their emotions and to manage their emotions may improve emotion regulation strategies [89]. However, these explanations must be tested further, and the results of the present study need to be replicated. Furthermore, given the variance in the model, there are clearly other risk factors, which were beyond the scope of this study but also need to be investigated.

When comparing both groups with each other, the results demonstrate that male users score higher in all dark triad traits. This partly supports previous research that showed males reported higher mean scores on Machiavellianism [41]. In contrast, previous research has reported that females scoring high on psychopathy were susceptible to greater levels of PSNSU [79]. The structural equation models for male and female participants however showed similar direct and mediation effects for both genders. Notably, the effect sizes were higher for female participants. Given that the main mechanism appears to be similar for male and female SNS users, the results emphasize interesting implications. For instance, they show that the key mechanisms described in the I-PACE model [18] appear to be valid for male and female SNS users, which suggest general validity. Affective and cognitive components such as reinforcement mechanisms of personality traits for the development of an addictive behaviour are similar for different groups. Furthermore, being younger in age also seems to be an additional risk factor. Future research should investigate the relevance of specific personality traits as well as further risk factors against the background of specific socio-demographic variables.

The present study is not without limitations. The cross-sectional nature of the study prevents the drawing of causal relationships. Furthermore, the study relied on self-report data which is subject to well-known biases including memory recall and social desirability. With the focus on dark triad traits, the factors of social desirability and impression management may have a negative impact on the reliability of the data [90, 91], such implications need to be addressed with more innovative research methods. The present study did not utilise a clinical sample, future research with similar aims amongst a clinical sample is therefore warranted. The study used a survey method, therefore future research could use mixed-methods to confirm or disconfirm the findings presented here. Investigating emotion regulation strategies (e.g., reappraisal, and suppression) in relation to SNS experiences using a qualitative approach would also be beneficial. Moreover,  regarding the age-sensitive constructs, it would be worth investigating the effect of age more systematically.

## Conclusions

The present study is the first to examine the possible mediation role of emotion dysregulation in the association between dark triad traits and PSNSU. The findings provide suggestive evidence of why PSNSU may develop as a function of the presence of dark triad traits and emotion dysregulation. The study highlighted the important role that emotion regulation plays in the association between dark triad traits and PSNSU. The findings will be of benefit to prevention and treatment programmes focusing on problematic online behaviours.

## Data Availability

The dataset used and analysed during the current study are available from the corresponding author on reasonable request.

## References

[1] Lenhart A (2015). Teens, social media, and technology overview 2015.

[2] Allen KA, Ryan T, Gray DL, McInerney DM, Waters L (2014). Social media use and social connectedness in adolescents: the positives and the potential pitfalls. Educ Dev Psychol.

[3] Siddiqui S, Singh T (2016). Social media its impact with positive and negative aspects. Int J Comput Appl Technol Res.

[4] Qualman E (2010). Socialnomics: how social media transforms the way we live and do business.

[5] Phua J, Jin SV, Kim JJ (2017). Uses and gratifications of social networking sites for bridging and bonding social capital: a comparison of Facebook, Twitter, Instagram, and Snapchat. Comput Hum Behav.

[6] Andreassen CS, Pallesen S (2014). Social network site addiction—an overview. Curr Pharm Des.

[7] World Health Organization. International Classification of Diseases 11th Revision. 2019. https://icd.who.int/en. Retrieved 17 Sept 2021.

[8] Brand M, Rumpf HJ, Demetrovics Z, Müller A, Stark R, King DL, Goudriaan AE, Mann K, Trotzke P, Fineberg NA, Chamberlain SR, Kraus SW, Wegmann E, Billieux J, Potenza MN (2020). Which conditions should be considered as disorders in the International Classification of Diseases (ICD-11) designation of “other specified disorders due to addictive behaviors”?. J Behav Addict.

[9] Koc M, Gulyagci S (2013). Facebook addiction among Turkish college students: the role of psychological health, demographic, and usage characteristics. Cyberpsychol Behav Soc Netw.

[10] Malik S, Khan M (2015). Impact of Facebook addiction on narcissistic behavior and self-esteem among students. J Pak Med Assoc.

[11] Tang CSK, Koh YYW (2017). Online social networking addiction among college students in Singapore: comorbidity with behavioral addiction and affective disorder. Asian J Psychiatry.

[12] Bányai F, Zsila A, Király O, Maraz A, Elekes Z, Griffiths MD, Andreassen CS, Demetrovics Z (2017). Problematic social media use: results from a large-scale nationally representative adolescent sample. PLoS ONE.

[13] Everitt BJ, Robbins TW (2016). Drug addiction: updating actions to habits to compulsions ten years on. Ann Rev Psychol.

[14] Everitt BJ, Robbins TW (2005). Neural systems of reinforcement for drug addiction: from actions to habits to compulsion. Nat Neurosci.

[15] Wegmann E, Mueller SM, Ostendorf S, Brand M (2018). Highlighting Internet-communication disorder as further Internet-use disorder when considering neuroimaging studies. Curr Behav Neurosci Rep.

[16] Carter BL, Tiffany ST (1999). Meta-analysis of cue-reactivity in addiction research. Addiction.

[17] Tiffany ST, Wray JM (2012). The clinical significance of drug craving. Ann New York Acad Sci.

[18] Brand M, Young KS, Laier C, Wölfling K, Potenza MN (2016). Integrating psychological and neurobiological considerations regarding the development and maintenance of specific Internet-use disorders: an Interaction of Person-Affect-Cognition-Execution (I-PACE) model. Neurosci Biobehav Rev.

[19] Brand M, Wegmann E, Stark R, Müller A, Wölfling K, Robbins TW, Potenza MN (2019). The Interaction of Person-Affect-Cognition-Execution (I-PACE) model for addictive behaviors: update, generalization to addictive behaviors beyond Internet-use disorders, and specification of the process character of addictive behaviors. Neurosci Biobehav Rev.

[20] Brand M, Laier C, Young KS (2014). Internet addiction: coping styles, expectancies, and treatment implications. Front Psychol.

[21] Wegmann E, Brand M (2016). Internet-communication disorder: it’s a matter of social aspects, coping, and Internet-use expectancies. Front Psychol.

[22] Caplan SE (2003). Preference for online social interaction: a theory of problematic Internet use and psychosocial well-being. Commun Res.

[23] Caplan SE (2010). Theory and measurement of generalized problematic Internet use: a two-step approach. Comput Hum Behav.

[24] Davis RA (2001). Cognitive-behavioral model of pathological Internet use. Comput Hum Behav.

[25] Kardefelt-Winther D (2014). A conceptual and methodological critique of internet addiction research: Towards a model of compensatory internet use. Comput Human Behav.

[26] Wegmann E, Brand M (2019). A narrative Overview About Psychosocial Characteristics as Risk Factors of a Problematic Social Networks Use. Current Addiction Reports.

[27] Andreassen CS (2015). Online social network site addiction: a comprehensive review. Curr Addict Rep.

[28] Andreassen CS, Griffiths MD, Gjertsen SR, Krossbakken E, Kvam S, Pallesen S (2013). The relationships between behavioral addictions and the five-factor model of personality. J Behav Addict.

[29] Andreassen CS, Torsheim T, Brunborg GS, Pallesen S (2012). Development of a Facebook addiction scale. Psychol Rep.

[30] Bianchi A, Phillips JG (2005). Psychological predictors of problem mobile phone use. Cyberpsychol Behav.

[31] Roberts JA, Pullig C, Manolis C (2015). I need my smartphone: a hierarchical model of personality and cell-phone addiction. Personal Individ Differ.

[32] Wang CW, Ho RT, Chan CL, Tse S (2015). Exploring personality characteristics of Chinese adolescents with internet-related addictive behaviors: trait differences for gaming addiction and social networking addiction. Addict Behav.

[33] Wang CW, Rainbow THH, Chan CLW, Tse S (2015). Exploring personality characteristics of Chinese adolescents with Internet-related addictive behaviors: trait differences for gaming addiction and social networking addiction. Addict Behav.

[34] Wilson K, Fornasier S, White KM (2010). Psychological predictors of young adults' use of social networking sites. Cyberpsychol Behav Soc Netw.

[35] Furnham A, Richards SC, Paulhus DL (2013). The Dark Triad of personality: a 10 year review. Soc Pers Psychol Compass.

[36] McCain JL, Borg ZG, Rothenberg AH, Churillo KM, Weiler P, Campbell WK (2016). Personality and selfies: narcissism and the Dark Triad. Comput Hum Behav.

[37] Smith MB, Hill AD, Wallace JC, Recendes T, Judge TA (2018). Upsides to dark and downsides to bright personality: a multidomain review and future research agenda. J Manag.

[38] Sindermann C, Sariyska R, Lachmann B, Brand M, Montag C (2018). Associations between the dark triad of personality and unspecified/specific forms of Internet-use disorder. J Behav Addict.

[39] Kircaburun K, Jonason PK, Griffiths MD (2018). The Dark Tetrad traits and problematic social media use: the mediating role of cyberbullying and cyberstalking. Personal Individ Differ.

[40] Demircioğlu ZI, Göncü Köse A (2021). Effects of attachment styles, dark triad, rejection sensitivity, and relationship satisfaction on social media addiction: a mediated model. Curr Psychol.

[41] Monacis L, Griffiths MD, Limone P, Sinatra M, Servidio R (2020). Selfitis behavior: assessing the Italian version of the selfitis behavior scale and its mediating role in the relationship of dark traits with social media addiction. Int J Environ Res Public Health.

[42] Thompson RA, Fox NA (1994). Emotion regulation: a theme in search of definition. The development of emotion regulation: biological and behavioural considerations.

[43] Gross JJ, Jazaieri H (2014). Emotion, emotion regulation, and psychopathology: an affective science perspective. Clin Psychol Sci.

[44] Sheppes G, Suri G, Gross JJ (2015). Emotion regulation and psychopathology. Annu Rev Clin Psychol.

[45] Estévez A, Jáuregui P, Sánchez I, Lopez-Gonzalez H, Griffiths MD (2017). Attachment and emotion regulation in substance and behavioural addictions. J Behav Addict.

[46] Tull MT, Bardeen JR, DiLillo D, Messman-Moore T, Gratz KL (2015). A prospective investigation of emotion dysregulation as a moderator of the relation between posttraumatic stress symptoms and substance use severity. J Anxiety Disord.

[47] Gratz KL, Roemer L (2008). The relationship between emotion dysregulation and deliberate self-harm among female undergraduate students at an urban commuter university. Cogn Behav Ther.

[48] Tull MT, Weiss NH, Adams CE, Gratz KL (2012). The contribution of emotion regulation difficulties to risky sexual behavior within a sample of patients in residential substance abuse treatment. Addict Behav.

[49] Ehring T, Tuschen-Caffier B, Schnülle J, Fischer S, Gross JJ (2010). Emotion regulation and vulnerability to depression: spontaneous versus instructed use of emotion suppression and reappraisal. Emotion.

[50] Hormes JM, Kearns B, Timko CA (2014). Craving Facebook? Behavioral addiction to online social networking and its association with emotion regulation deficits. Addiction.

[51] Elhai JD, Tiamiyu MF, Weeks JW, Levine JC, Picard KJ, Hall BJ (2018). Depression and emotion regulation predict objective smartphone use measured over one week. Personal Individ Differ.

[52] Casale S, Caplan SE, Fioravanti G (2016). Positive metacognitions about Internet use: the mediating role in the relationship between emotional dysregulation and problematic use. Addict Behav.

[53] Spada MM, Marino C (2017). Metacognitions and emotion regulation as predictors of problematic internet use in adolescents. Clin Neuropsychiatry.

[54] Kircaburun K, Alhabash S, Tosuntaş ŞB, Griffiths MD (2019). Uses and gratifications of problematic social media use among university students: a simultaneous examination of the Big Five of personality traits, social media platforms, and social media use motives. Int J Ment Health Addict.

[55] Andreassen CS, Billieux J, Griffiths MD, Kuss DJ, Demetrovics Z, Mazzoni E, Pallesen S (2016). The relationship between addictive use of social media and video games and symptoms of psychiatric disorders: a large-scale cross-sectional study. Psychol Addict Behav.

[56] Lin C-Y, Broström A, Nilsen P, Griffiths MD, Pakpour AH (2017). Psychometric validation of the Persian Bergen Social Media Addiction Scale using classic test theory and Rasch Models. J Behav Addict.

[57] Monacis L, de Palo V, Griffiths MD, Sinatra M (2017). Social networking addiction, attachment style, and validation of the Italian version of the Bergen Social Media Addiction Scale. J Behav Addict.

[58] Yam C-Y, Pakpour A, Griffiths MD, Yau W-Y, Lo C-YM, Jennifer MT, Ng JMT, Lin CY, Leung H (2019). Psychometric testing of three Chinese online-related addictive behavior instruments among Hong Kong university students. Psychiatr Q.

[59] Griffiths M (2005). A ‘components’ model of addiction within a biopsychosocial framework. J Subst Use.

[60] Satici SA (2018). Facebook addiction and subjective well-being: a study of the mediating role of shyness and loneliness. Int J Ment Health Addict.

[61] Satici SA, Uysal R (2015). Well-being and problematic Facebook use. Comput Hum Behav.

[62] Brailovskaia J, Rohmann E, Bierhoff HW, Margraf J, Köllner V (2019). Relationships between addictive Facebook use, depressiveness, insomnia, and positive mental health in an inpatient sample: A German longitudinal study. J Behav Addict.

[63] Jones DN, Paulhus DL (2014). Introducing the short dark triad (SD3) a brief measure of dark personality traits. Assessment.

[64] Bjureberg J, Ljótsson B, Tull MT, Hedman E, Sahlin H, Lundh LG, Bjärehed J, DiLillo D, Messman-Moore T, Gumpert CH, Gratz KL (2016). Development and validation of a brief version of the difficulties in emotion regulation scale: the DERS-16. J Psychopathol Behav Assess.

[65] Muthén L, Muthén B (2011). MPlus.

[66] Little TD, Cunningham WA, Shahar G, Widaman KF (2002). To parcel or not to parcel: exploring the question, weighing the merits. Struct Equ Model.

[67] Marsh HW, Lüdtke O, Nagengast B, Morin AJ, von Davier M (2013). Why item parcels are (almost) never appropriate: two wrongs do not make a right camouflaging misspecification with item parcels in CFA models. Psychol Methods.

[68] Hu L, Bentler PM, Hoyle RH (1995). Evaluating model fit. Structural equation modeling concepts issues and applications.

[69] Hu L, Bentler PM (1999). Cutoff criteria for fit indexes in covariance structure analysis: conventional criteria versus new alternatives. Struct Equ Model.

[70] Baron RM, Kenny DA (1986). The moderator–mediator variable distinction in social psychological research: conceptual, strategic, and statistical considerations. J Pers Soc Psychol.

[71] Ostendorf S, Wegmann E, Brand M (2020). Problematic social-networks-use in German children and adolescents—the interaction of need to belong, online self-regulative competences, and age. Int J Environ Res Public Health.

[72] Shensa A, Escobar-Viera CG, Sidani JE, Bowman ND, Marshal MP, Primack BA (2017). Problematic social media use and depressive symptoms among US young adults: a nationally-representative study. Soc Sci Med.

[73] Dimitrov DM (2006). Comparing groups on latent variables: a structural equation modeling approach. Work.

[74] Ünal-Aydın P, Balıkçı K, Sönmez İ, Aydın O (2020). Associations between emotion recognition and social networking site addiction. Psychiatry Res.

[75] Ünal-Aydın P, Obuća F, Aydın O, Spada MM (2021). The role of metacognitions and emotion recognition in problematic SNS use among adolescents. J Affect Disord.

[76] Fox J, Rooney MC (2015). The Dark Triad and trait self-objectification as predictors of men’s use and self-presentation behaviors on social networking sites. Personal Individ Differ.

[77] Hussain Z, Griffiths MD, Sheffield D (2017). An investigation into problematic smartphone use: the role of narcissism, anxiety, and personality factors. J Behav Addict.

[78] Pearson C, Hussain Z (2015). Smartphone use, addiction, narcissism, and personality: a mixed methods investigation. Int J Cyber Behav Psychol Learn.

[79] Chung KL, Morshidi I, Yoong LC, Thian KN (2019). The role of the dark tetrad and impulsivity in social media addiction: findings from Malaysia. Personal Individ Differ.

[80] Krämer NC, Winter S (2008). Impression management 2.0: the relationship of self-esteem, extraversion, self-efficacy, and self-presentation within social networking sites. J Media Psychol.

[81] Ong EYL, Ang RP, Ho JCM, Lim JCY, Goh DH, Lee CS, Chua AYK (2011). Narcissism, extraversion and adolescents’ self-presentation on Facebook. Personal Individ Differ.

[82] Walther JB (2007). Selective self-presentation in computer-mediated communication: hyperpersonal dimensions of technology, language, and cognition. Comput Hum Behav.

[83] Michikyan M, Subrahmanyam K, Dennis J (2014). Can you tell who I am? Neuroticism, extraversion, and online self-presentation among young adults. Comput Hum Behav.

[84] Balcerowska J, Biernatowska A, Golińska P, Barańska J (2019). Relationship between dimensions of grandiose narcissism and Facebook addiction among university students. Curr Issues Pers Psychol.

[85] Casale S, Banchi V (2020). Narcissism and problematic social media use: a systematic literature review. Addict Behav Rep.

[86] Paulhus DL, Williams KM (2002). The Dark Triad of personality: narcissism, Machiavellianism, and psychopathy. J Res Pers.

[87] Dong G, Potenza MN (2014). A cognitive-behavioral model of Internet gaming disorder: theoretical underpinnings and clinical implications. J Psychiatr Res.

[88] Servidio R, Sinatra M, Griffiths MD, Monacis L (2021). Social comparison orientation and fear of missing out as mediators between self-concept clarity and problematic smartphone use. Addict Behav.

[89] Karreman A, Vingerhoets AJ (2012). Attachment and well-being: the mediating role of emotion regulation and resilience. Personal Individ Differ.

[90] Keillor BD, Owens D, Pettijohn C (2001). A cross-cultural/cross national study of influencing factors and socially desirable response biases. Int J Mark Res.

[91] Kowalski CM, Rogoza R, Vernon PA, Schermer JA (2018). The Dark Triad and the self-presentation variables of socially desirable responding and self-monitoring. Personal Individ Differ.

